# The Human Metapneumovirus Matrix Protein Stimulates the Inflammatory Immune Response *In Vitro*

**DOI:** 10.1371/journal.pone.0017818

**Published:** 2011-03-11

**Authors:** Audrey Bagnaud-Baule, Olivier Reynard, Magali Perret, Jean-Luc Berland, Mimoun Maache, Christophe Peyrefitte, Guy Vernet, Viktor Volchkov, Gláucia Paranhos-Baccalà

**Affiliations:** 1 bioMérieux, Emerging Pathogens Department, Institut Fédératif de Recherche 128 BioSciences Lyon Gerland, Lyon, France; 2 Fondation Mérieux, Laboratoire des Pathogènes Emergents, Lyon Gerland, Lyon, France; 3 INSERM U758 Biologie des Filovirus, IFR128 BioSciences Lyon Gerland, Lyon, France; 4 Institut de Recherche Biomédicale des Armées, Grenoble, France; National Institute for Infectious Diseases (L. Spallanzani), Italy

## Abstract

Each year, during winter months, human Metapneumovirus (hMPV) is associated with epidemics of bronchiolitis resulting in the hospitalization of many infants. Bronchiolitis is an acute illness of the lower respiratory tract with a consequent inflammation of the bronchioles. The rapid onset of inflammation suggests the innate immune response may have a role to play in the pathogenesis of this hMPV infection. Since, the matrix protein is one of the most abundant proteins in the *Paramyxoviridae* family virion, we hypothesized that the inflammatory modulation observed in hMPV infected patients may be partly associated with the matrix protein (M-hMPV) response. By western blot analysis, we detected a soluble form of M-hMPV released from hMPV infected cell as well as from M-hMPV transfected HEK 293T cells suggesting that M-hMPV may be directly in contact with antigen presenting cells (APCs) during the course of infection. Moreover, flow cytometry and confocal microscopy allowed determining that M-hMPV was taken up by dendritic cells (moDCs) and macrophages inducing their activation. Furthermore, these moDCs enter into a maturation process inducing the secretion of a broad range of inflammatory cytokines when exposed to M-hMPV. Additionally, M-hMPV activated DCs were shown to stimulate IL-2 and IFN-γ production by allogeneic T lymphocytes. This M-hMPV-mediated activation and antigen presentation of APCs may in part explain the marked inflammatory immune response observed in pathology induced by hMPV in patients.

## Introduction

Human Metapneumovirus (hMPV), a member of the *Pneumoviridae* subfamily of *Paramyxoviridae*, has been recognized as a leading cause of acute respiratory disease in children worldwide [1]. Studies have shown that hMPV infects approximately half of all infants under the age of 1 year and that this figure increases to virtually all children by the age of 10 [2], [3]. Clinically, hMPV-associated disease includes rhinitis, pharyngitis, bronchitis, bronchiolitis and pneumonia, and resembles that of human respiratory syncytial virus (RSV) infection [4]–[6]. However, the immune mechanisms and viral pathogenesis of respiratory illness related to hMPV infection are not well understood and many questions remain unanswered. Nevertheless, a widely accepted hypothesis is that the pathogenesis of viral infection in the lower respiratory tract of infants is due to the induction of innate inflammatory responses [7]–[9]. This is supported by the observation that inflammatory cytokines are detected in the nasopharyngeal and the tracheal secretions of children with respiratory disease induced by viral infection [10].

DCs are the most influential APC [11] residing in nearly all peripheral tissues, including the respiratory tract, and are vital in the early response to pathogens [12], such as the paramyxovirus hMPV. Similarly, alveolar macrophages which are also APCs, are capable of inducing a pro-inflammatory cascade in response to viruses [13]. With this in mind, understanding the outcomes of interactions between viruses, such as hMPV, and mucosal APCs is critical to understand disease pathogenesis.

A characteristic of the paramyxovirus family is that they have a lipid envelope containing two membrane glycoproteins: an attachment glycoprotein (G) and a fusion protein (F). The nucleocapsid core contains the viral RNA genome as well as the nucleocapsid (N), phospho- (P), and large (L) proteins [14]. Between the envelope and the core resides what has been described as one of the most abundant proteins of the virion: the viral matrix (M) protein [15]. The M protein appears to play two key roles: firstly, it inhibits the transcriptase activity of the nucleocapsid prior to packaging and, secondly, the M protein mediates the association of the nucleocapsid with the cell plasma membrane [15]–[18]. All paramyxovirus M proteins are known to have membrane associations and similar hydropathy profiles and are presumed to play a crucial role in virus assembly and budding [19]. The M protein of RSV has been observed in HEp2 infected cells [20] throughout the cells and in cytoplasmic inclusions. It suggests that this M protein can be released from these infected cells in case of cell death or by an active process. M-hMPV may then possibly generate signals that influence the immune response. To date, M protein functions, have been described [20]–[22] but the role played by these M protein in the immune process needs further investigations, particularly in regard to the modulation of the immune response.

In the present study, we show that the M-hMPV protein can be released in a soluble form and *in vitro*, human macrophages and DCs can be activated by the recombinant M-hMPV protein. Our findings provide new insights about the role of M-hMPV protein in the modulation of the immune response to virus and may provide information to support a better understanding of hMPV pathogenesis.

## Materials and Methods

### Viral preparation

The hMPV strain stock NL/1/00 corresponding to supernatant and cells was obtained from Pr. A.D.M.E Osterhaus and Dr. J. Simon at ViroNovative B.V., Rotterdam, The Netherlands. Briefly, Vero cells were infected with the hMPV/NL/1/00 [1] at MOI 1, incubated at 34°C and cultured in IMDM (minimal essential medium) (Invitrogen) in the presence of glutamine (2 mM), 0.3% BSA, 0.000375% trypsin, penicillin 100 U/mL and streptomycin 100 µg/mL (Invitrogen). Vero cells were maintained in IMDM supplemented with 10% fetal calf serum (FCS), 2 mM L-glutamine, penicillin 100 U/mL and streptomycin 100 µg/mL antibiotics (all from Invitrogen).

### M-hMPV protein capture experiment

M-hMPV protein was detected from supernatant of hMPV infected Vero cells using positively charged magnetic beads obtained from Dr. R. Veyret, bioMérieux, Lyon, France [23]. Briefly, 1 mL of infected Vero cells supernatant was recovered after 11 days of infection (first observation of the cytopathic effect), ultracentrifugated (250 000 rpm at 4°C for 3 h in a Beckman SW41 rotor ultracentrifuge) and then incubated with 10 µg of magnetic beads for 10 min. Magnetic beads were then recovered by using a magnet and the preparation was analyzed by western blot.

### Western blotting

Western blotting was performed by using 1 µg/mL 8D12F6 and 1D7A7 monoclonal antibodies directed against M-hMPV (in house validated antibodies, bioMérieux). The membrane was washed 10 min, three times in PBS-T and then incubated for 1 h at room temperature with 1 µg/mL goat anti-mouse immunoglobulin G horseradish peroxydase antibody (Promega). After three washes in PBS-T for 10 min each time, the membrane was developed by ECL-Western blotting detection reagents (AP Biotech, Piscataway, NJ) according to the manufacturer's instructions and exposed to Kodak X-OMAT film for 45 min.

### Expression and purification of hMPV matrix protein in bacteria

cDNA was obtained from a RNA sample extracted using the Nuclisens kit (bioMérieux) from nasal swab of children mono-infected by hMPV genotype A1 and hospitalized with bronchiolitis. The M-hMPV (nucleotides 2165–2929) was expressed in *E. coli* and purified as described previously [24]. The amplification of the M protein genomic sequence corresponding to amino acids 1–253 was achieved by reverse transcriptase-PCR (RT-PCR). Briefly, the RT step was performed with primer 5′-ATGGAGTCCTACCTAGTAGA-3′ using Thermoscript RT kit (Invitrogen) (70°C–8 min, 58°–1 h, 95°C–5 min, 4°C) followed by two complementary PCR assays using the taq polymerase high fidelity kit with primers 5′-ATGGAGTCCTACCTAGTAGA-3′ and 5′-TCTGGACTTCAAGACATATC-3′ (95°C–2 min, 95°C–30 sec, 52°C–1 min, 68°C–1 min, 68°C–7 min, 4°C for 30 cycles) and 5′-CTCTCTGCTAGCGAGTCCTA CCTAGTAGACACCTATCAAGG-3′C and 5′-CTCTCTGGATCCTTAGTGGT GATGGTGATGGTGAGAACCC CTCATTCTGGACCTCAAGAC ATATCT-3′, (95°C–2 min, 95°C–30 sec, 60°C–1 min, 68°C–1 min, 68°C–7 min, 4°C for 30 cycles). These primers introduce a MRGS-6xHis tag at the C-terminal end. The *Nhe*I and *Bam*HI (underlined) digested PCR products were ligated into the digested pET21-b expression vector (Novagen). *E. coli* BL21DE3 bacteria containing recombinant pET21-b/M-hMPV was grown until the OD_600_ reached 0.6 and induced with 0.4 mM isopropyl-beta-D-thiogalactopyranoside. After 3 h, bacteria cells were centrifuged and resuspended in a lysis buffer, disrupted by sonication (3×10 s–30 watts), followed by further centrifugation. M-hMPV protein was absent from supernatant and was then extracted from pelleted bacteria by a 6M urea buffer. This was then applied on Ni-NTA Agarose (Qiagen) for purification according to the manufacturer's instructions in endotoxin-free conditions. 6xHis-tagged recombinant M-hMPV protein was eluted from the column by a pH gradient. Analysis of eluted fraction was performed with Coomassie brilliant blue staining after electrophoresis on 12% SDS-polyacrylamide gel under reducing conditions.

Mass spectrometry analysis was completed using MALDI-TOF in a voyager-DE-PRO (Applied Bio- systems, CA, USA) and only the M-hMPV protein was observed.

The endotoxins concentration of the purified M-hMPV was determined by a kinetic chromogenic-QCL Bio Wittaker Limulus amoebocyte lysate assay at Laboratoire Marcel Mérieux (Lyon, France) and was negative (<0.25 U endotoxins/mg of M-hMPV protein). The RNA contaminants detection was performed by RNA Array 6000 Pico kit from Agilent (Agilent Technologies) and by detection of RNA 16S with universal PCR Light Cycler Fast Start Master SYBR Green I. The protein M-hMPV preparation did not contain RNA contaminants. The M-hMPV protein was labeled by using Fluorescein-NHS (Pierce) and the preparation controlled for the absence of endotoxins. Free fluorescein was removed by dialysis and labeled protein was filtered at 22 µM and recovered in PBS/SDS 0.01%/Azide 0.9 g/L.

### Molecular cloning of eukaryotic M-hMPV protein and transfection of HEK 293T cells

The M-hMPV coding sequence was cloned into the vector phCMV in *Bam*HI restriction site. M-hMPV sequence was amplified from bacterial expression plasmid pET21b/M-hMPV by PCR using primer introducing BamHI restriction site: 5′-CTAGGATCCCACCATGGCTAGCGAGTCCTAC-3′ and 5′-CTAGGATCCTTATCTGGACTTCAAGACATATC-3′. phCMV/M-hMPV was transfected in HEK 293T cells [25]. HEK 293T cells were cultured at 37°C in Dulbecco's modified Eagle's medium (Invitrogen) supplemented with 10% FCS (Perbio). Cells were grown in 75 cm^2^ flask to a confluence of 60%. Transfection was performed using FuGENE HD (Roche) according to the manufacturer's instructions. Briefly, 10 µg of DNA was diluted in 1000 µL of serum-free DMEM, and 30 µL of FuGENE HD was added to the diluted DNA. After 15 min of incubation, the mixture was added to the cells and incubated for 6 h at 37°C. Then, 30 mL of DMEM supplemented with 10% FCS was added to the cells.

### M protein release assay and production of soluble M-hMPV in eukaryotic cells

48 h after transfection, the cells supernatant was harvested and centrifuged (5 min, 1 000 *g*, 4°C) to remove cellular debris. The clarified supernatant was loaded on a 20% sucrose cushion and centrifuged at 250 000 rpm at 4°C for 3 h in a Beckman SW41 rotor ultracentrifuge. After ultracentrifugation the upper phase containing soluble protein was harvested and the pellet was resuspended in 1 mL of PBS.

Flotation assay. Supernatant of M-hMPV transfected cells was adjusted to 60% histodenz (Sigma) wt/wt, loaded at the bottom of a tube, and layered with 8 mL 40% histodenz and 2 mL of 10% histodenz. Tubes were centrifuged for 20 h at 250 000 rpm in a Beckman SW41 rotor.

### Generation of monocyte-derived DCs (moDCs) and monocyte-derived macrophages (MDMs)

Human peripheral blood from healthy donors was obtained from the Etablissement Français du Sang (Lyon, France). Mononuclear cells were isolated by density gradient centrifugation using Ficoll-Hypaque, and then centrifuged on a 50% Percoll solution (Amersham Biosciences). Two fractions were recovered: monocytes were purified from the light density fraction and T lymphocytes from the high density fraction. The purification of monocytes and lymphocytes were performed by immunomagnetic beads depletion (Dynal Biotech) using a cocktail of monoclonal antibodies (mAb): anti-CD3, anti-CD16, anti-CD19, anti-CD56 and anti-glycophorin A for monocytes purification and anti-CD14, anti-CD16, anti-CD19, anti-CD56, and anti-glycophorin A for lymphocytes purification (all monoclonal antibodies from Beckman Coulter). Monocytes were >95% enriched as assessed by CD14 labeling and T lymphocytes >95% pure as assessed by CD3 labeling. Monocytes (10^6^ cells/mL) were differentiated to immature DCs (iDCs) and macrophages (MDMs Macrophage-derived monocytes) in complete RPMI 1640 medium containing 2 mM glutamine, 10 mM HEPES, 40 ng/mL gentamicin (all from Life Technologies) and 10% FCS (Biowest), supplemented with 40 ng/mL human recombinant granulocyte/macrophage colony-stimulating factor (GM-CSF) plus 250 U/mL human recombinant IL-4 for iDCs or 40 ng/mL macrophage colony-stimulating factor (M-CSF) for MDMs (all from Abcys) and incubated for 5 days at 37°C in 5% CO_2_.

### M-hMPV binding and internalization

The binding of Fluorescein-M-hMPV was performed on differentiated cells harvested on day 5 and resuspended in culture medium. Different concentrations of Fluorescein-M-hMPV were incubated with cells (10^6^/mL) for 20 min on ice. Cells were then washed and recovered in cold FACS buffer. In competition experiments, 4.1 nM M-hMPV-Fluorescein was incubated with cells during a 20 min period on ice, washed twice by cold medium and an increased non-labeled concentration of M-hMPV was then added to the cells and incubated on ice for 20 min. Those cells were then washed and recovered in cold FACS buffer. To follow receptor-mediated endocytosis, cells were incubated on ice for 20 min with 1.3 nM Fluorescein-M-hMPV, washed twice at 4°C, and then incubated at 37°C for 5 to 30 min in complete RPMI medium. Internalization was stopped on ice with cold PBS containing 0.1% BSA and 0.05% NaN_3_. 2×10^5^ cells of binding and internalization experiments were added per round 12 mm diameter coverslip (Marienfeld) coated with poly-l-lysine (Sigma) and incubated 1 h at 4°C for binding and at 37°C for internalization. Cells were fixed using 4% paraformaldehyde (PFA) for 10 min, rinsed with PBS and examined by confocal microscopy using a non inverted Axioplan2 LSM510 Zeiss, as well as the Zeiss LSM Image Browser software (Zeiss).

### APCs phenotypic response to M-hMPV

To determine its effect, the M-hMPV recombinant protein was added to the iDCs for 24 h. We tested the bacterial recombinant M-hMPV protein as well as different protein controls: the protein elution buffer, the protein treated by polymyxin B sulphate (10 µg/mL) (Sigma), and heated at 20 min at 100°C. Also, LPS (10 ng/mL) (from *E. coli* isotype 0111:B4) (Sigma) was used as a positive control of phenotype maturation and HCV-NS3 Helicase recombinant protein (HCV-NS3H) produced in the same conditions as the M-hMPV protein as a negative control.

After 24 h of treatment, cell phenotype was analyzed by flow cytometry on a FACS Calibur (BD Biosciences) using CELLQUEST software (Becton Dickinson) (10 000 events/test). FITC-conjugated anti-CD14, -HLA-DR, -CD80, and phycoerythrin (PE)-conjugated anti-CD1a, -CD86, -CD83 and -CD40 were used to phenotype the cells (all from Immunotech). An isotypic control was also used to set the gating for the cells.

### Apoptosis measurement

1×10^6^ moDCs were pelleted, re-suspended in 100 µL of PBS containing 40 nM 3,3′-dihexyloxacarbocyanine iodide (DiOC6(3) (Molecular Probes) and incubating 30 min at room temperature in the dark. After incubation, cells were washed twice in PBS and re-suspended in FACS buffer. The DiOC6(3) membrane potential-related fluorescence was analyzed by flow cytometry on a FACS Calibur.

Quantification of apoptotic nuclei was also assessed by staining apoptotic nuclei with Propidium Iodide. Briefly, 25 µM of PI was added to the 1×10^6^ DiOC6(3) labelled cells prior analysis by flow cytometry.

### Cytokine production by APCs in response to M-hMPV protein

Supernatants from moDCs were assayed for cytokines IL-12p70, TNF, IL-10, IL-6, IL-1β, and IL-8 and supernatants from MDMs were assayed for chemokines MIP-1β, RANTES and TNF by Cytometric Bead Array (CBA, BD Biosciences). IFN-α and IFN-β were measured using ELISA kits (PBL Biomedical Laboratories).

Mixed lymphocyte reaction (MLR). Naive T-lymphocytes were purified from human peripheral blood as previously described in section “Generation of monocyte-derived DCs (moDCs) and monocyte-derived macrophages (MDMs)”. The cell assays were carried out in 96-well flat-bottom culture plates in triplicate for each condition. iDCs were treated for 24 h with LPS (positive control), HCV-NS3H protein (negative control), elution buffer and M-hMPV protein. After incubation at 37°C in 5% CO_2_, cells were collected, extensively washed, resuspended in RPMI 10% FCS and then co-cultured with 2×10^5^ allogeneic T-cells in 200 µL of complete culture medium at 1∶5, 1∶10 or 1∶20 APC: responder T-cell ratio. After co-culture at 37°C in 5% CO_2_, supernatants were recovered and tested for an IL-2 (day 2), IL-5, IL-13, and IFN-γ (day 5) secretion assay by Cytometric Bead Array flex (BD Biosciences).

## Results

### M-hMPV protein detection in the supernatant

**Figure 1:**
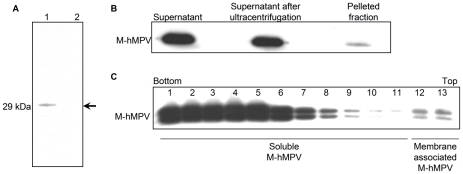
M-hMPV protein is released both in the supernatant of hMPV infected cells and M-hMPV transiently transfected cells. (A) M-hMPV protein was captured in the supernatant of the infected Vero cells by using positively charged magnetic beads. Samples were incubated with 1 µg/mL monoclonal antibodies directed against M-hMPV and 1 µg/mL goat anti-mouse immunoglobulin G antibody conjugated to horseradish peroxidase antibodies and revealed by ECL susbtrate. 1: ultracentrifugated supernatant of hMPV infected Vero cells; 2: supernatant of uninfected Vero cells. (B) 293T cells were transiently transfected by phCMV/M-hMPV. After 48 h, the supernatant was subjected to ultracentrifugation over a 20% sucrose cushions. The pelleted material was resuspended in PBS. Samples were analyzed by western blot in reducing condition using anti M-hMPV antibodies. (C) A flotation assay on the supernatant of M-hMPV transfected 293T cells was performed. Fractions 1 to 5 represent sample fraction in 60% histodenz, fraction 6 to 11, 40% histodenz and fraction 12 and 13, 10% histodenz. Membrane associated proteins are found at the interface of 40% and 10%. Fractions were analyzed by western blot in non reducing condition by using anti M-hMPV antibodies.

We hypothesized that M-hMPV can be released from hMPV infected cells. To confirm this hypothesis, we captured the protein from the supernatant of hMPV infected Vero cells. After ultracentrifugation to remove virus particles, anionic charge polymers beads were used to capture the free soluble M-hMPV (figure 1A) confirming that M-hMPV can be released during hMPV cell infection.

To support the previous result, 293T cells were transiently transfected by the vector phCMV encoding M-hMPV. A soluble form of M-hMPV in the supernatant of transfected cells was detected and analyzed. As seen in figure 1B, more than 90% of the M-hMPV was detected in the supernatant and this was further confirmed by the observation that after ultracentrifugation the M-hMPV protein was still identified in the supernatant. Furthermore, a lower amount was found in the pellet fraction. To establish whether the M-hMPV protein was associated with the cell membrane, we performed a flotation assay using the total cell supernatant. As shown in figure 1C, most of the M-hMPV did not float and stayed towards the bottom of the tube indicating that the protein was generally not associated with cell membranes. However, a small amount of the M protein was found in the upper phase suggesting the presence of a possible M-hMPV form that interacts with membranes. Under non-reducing conditions, M-hMPV displayed a double band pattern indicating a possible internal disulfide bridge.

### M-hMPV binds APCs

**Figure 2:**
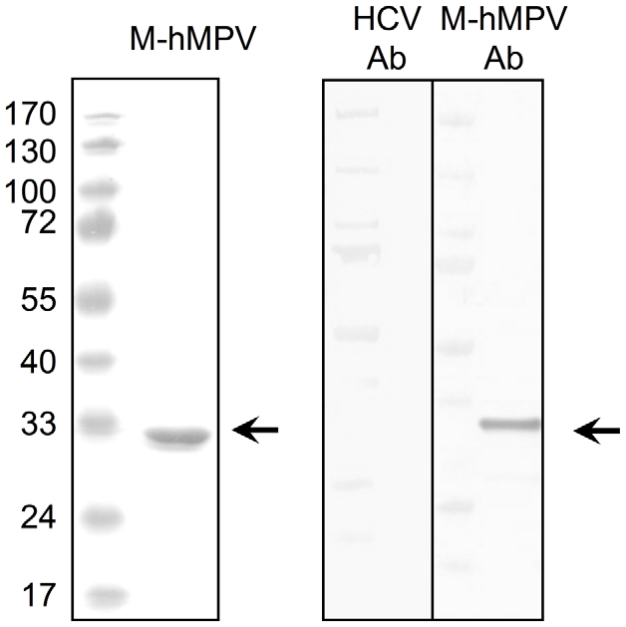
M-hMPV bacterial recombinant protein is successfully produced. (A) SDS-PAGE analysis of M-hMPV recombinant protein was performed after purification by Ni-NTA resin. (B) M-hMPV protein was analyzed by western blot using M-hMPV 1 µg/mL monoclonal antibodies and HCV monoclonal antibody for the negative control together with species-specific secondary antibody goat anti-mouse IgG H+L labeled with alkaline phosphatase.

**Figure 3:**
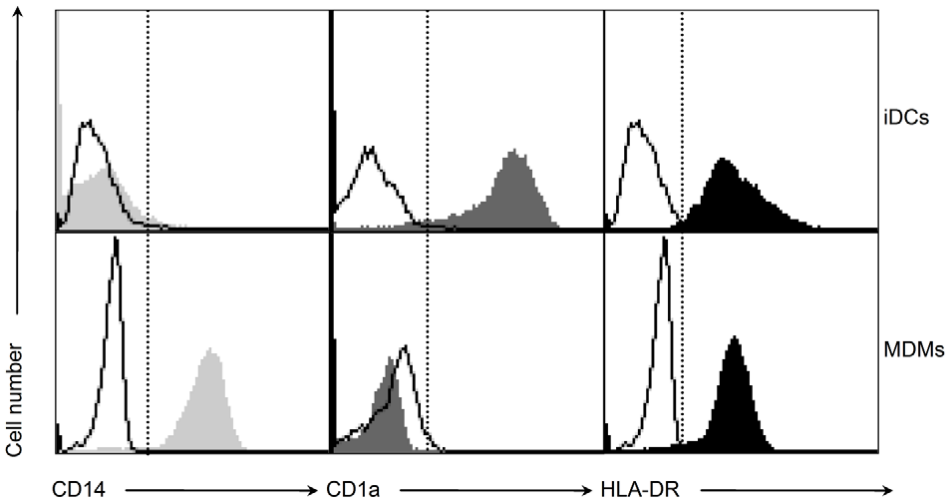
MoDCs express the CD1a molecule while MDMs express the CD14 molecule. Monocytes were differentiated during 5 days in complete RPMI medium containing 40 ng/mL GM- CSF and 250 U/mL IL-4 for moDCs and 40 ng/mL M-CSF for MDMs. Phenotype of cells were analyzed on day 5 for the expression of CD14, CD1a and HLA-DR. Data represent histograms with isotype control (bold line) and monocyte-differentiated cells (filled profile).

**Figure 4:**
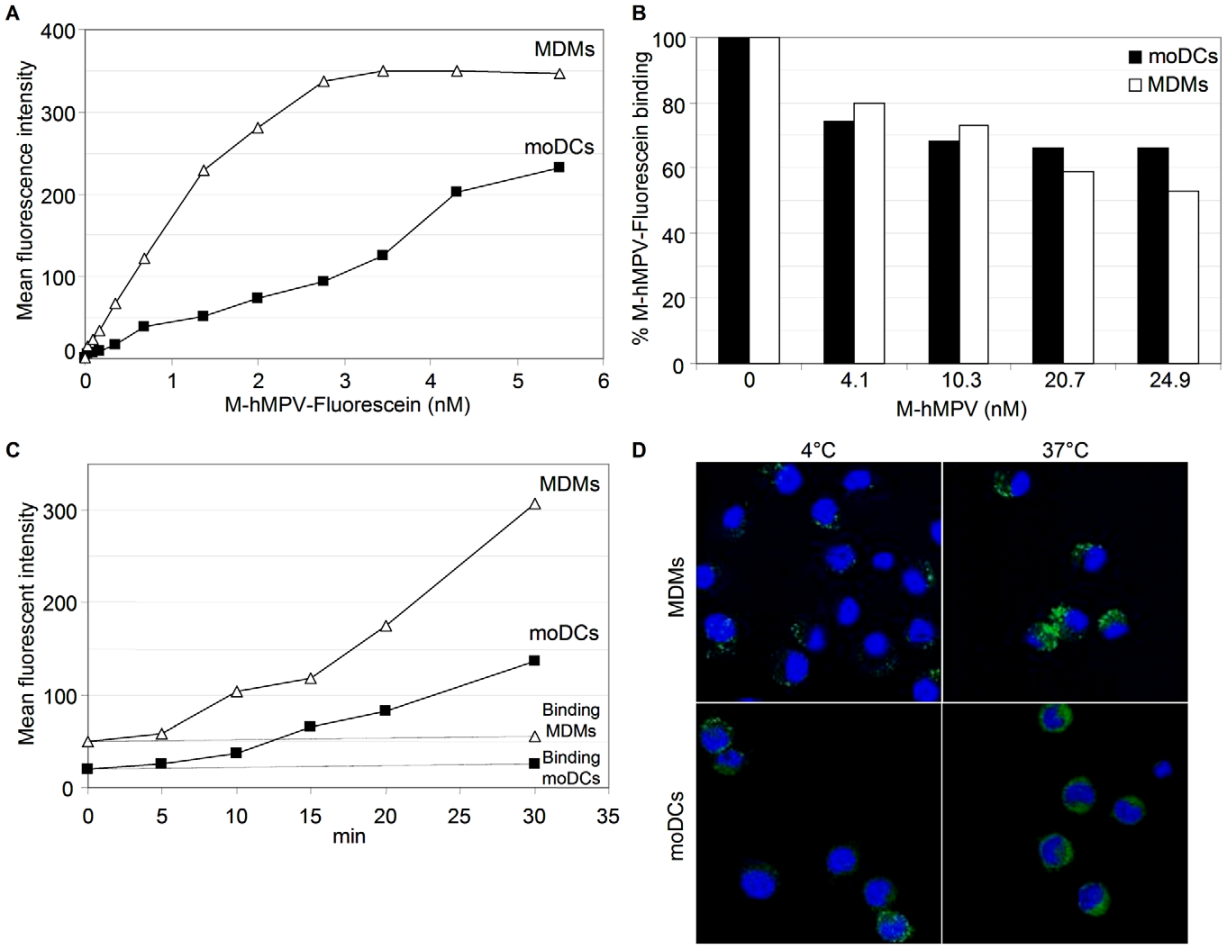
M-hMPV protein binds APCs and is internalized. (A) Binding experiment. APCs were incubated with increased concentrations of M-hMPV-Fluorescein protein for 20 min at 4°C, washed and then analyzed by FACS. Data are shown as mean fluorescence intensity. (B) Competition experiment. APCs were incubated with M-hMPV-Fluorescein protein 4.1 nM for 20 min at 4°C, washed before addition of increased concentration of unlabeled M-hMPV protein for 20 min at 4°C. Results are shown as % of mean fluorescent intensity of M-hMPV-Fluorescein protein binding. (C) Internalization experiment. Cells were incubated with 1.3 nM M-hMPV-Fluorescein protein during 20 min at 4°C, washed and incubated at different time points at 37°C. Cells were then washed and analyzed. Data are shown as mean fluorescence intensity. (D) Confocal microscopy. Cells were incubated with 0.17 nM M-hMPV protein 30 min at 4°C and then washed and incubated or not at 37°C for 30 min. Observation was realized by confocal microscopy. Data represent one out of two experiments.

Since M-hMPV is released, we investigated the potential of this protein to interact with APCs. We produced and used an endotoxin-free prokaryotic recombinant M-hMPV protein (figure 2). APCs were generated *in vitro* by cultivating human monocytes with differentiation factors. Analysis of the moDCs phenotype showed that at day 5 of culture, these cells had little expression of CD14 but high levels of CD1a and intermediate levels of HLA-DR (figure 3). Conversely, MDMs displayed high expression of CD14 and little expression of CD1a and the same intermediate expression of HLA-DR. To determine the uptake of the M-hMPV protein by moDCs, we analyzed the binding of M-hMPV labeled with fluorescein to moDCs and to MDMs by flow cytometry. M-hMPV bound moDCs and MDMs (figure 4A) and this binding was dose-dependent, partially saturable for moDCs and completely saturable for MDMs at a concentration of 2.7 nM. When fluorescent-M-hMPV binding was competed using an unlabeled M-hMPV at 4°C, M-hMPV was bound more efficiently in MDMs than in moDCs (figure 4B) indicating that a receptor may be implicated in the binding process and those MDMs had possibly more receptors than moDCs. From this, fluorescent M-hMPV appeared to be efficiently and rapidly internalized after the binding at 4°C and the incubation of the cells at 37°C (figure 4C). To further investigate the uptake of M-hMPV, confocal microscopy (figure 4D) was used to visualize the interaction with APCs. Analysis of the samples of APCs cultured with fluorescent M-hMPV showed isolated clusters for MDMs as compared to a diffuse labeling in moDCs. All together, these data suggest the idea that M-hMPV may bind specifically to the surface of MDMs. In contrast, for moDCs the data suggested the existence of different receptors involved in the binding profile.

### M-hMPV induces moDCs maturation

**Figure 5:**
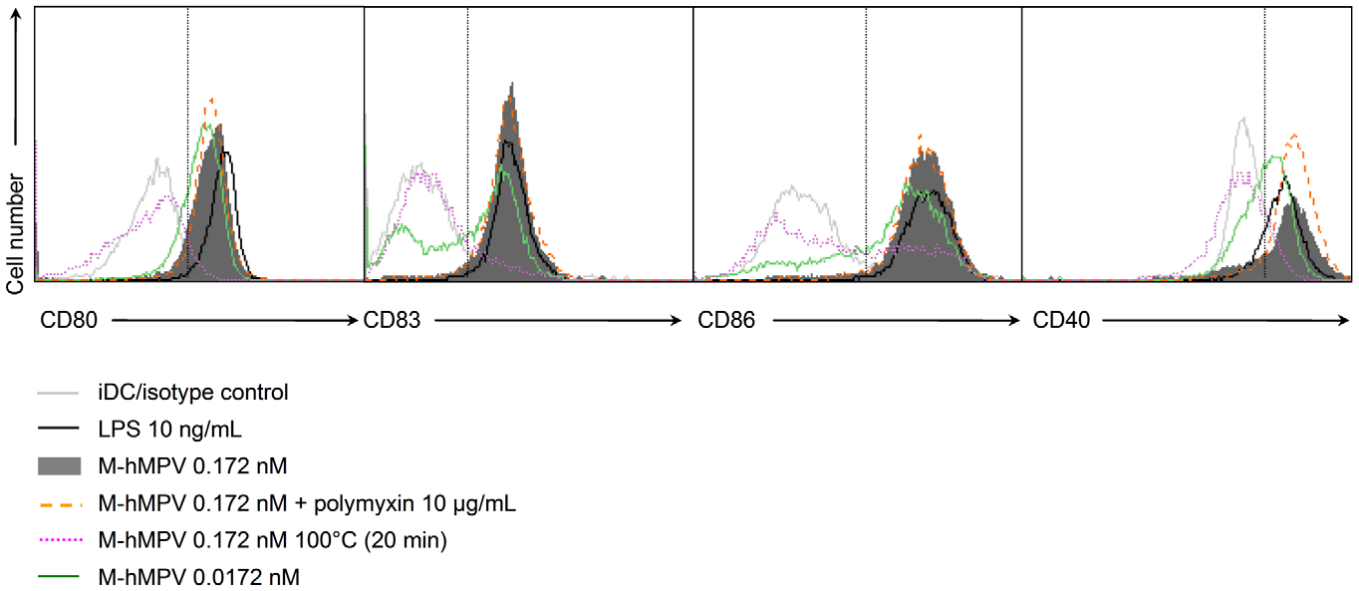
M-hMPV protein induces maturation of moDCs. moDCs were treated with M-hMPV protein 0.172 nM (gray filled) and 0.0172 nM (green line), LPS 10 ng/mL (black line), M-hMPV 0.172 nM+polymyxin (orange line), M-hMPV heat treated at 100°C–20 min (pink dotted line) or untreated (iDCs) (gray line) and the phenotype was analyzed after 24 h of treatment. Cells were stained with labeled CD80, CD83, CD86 and CD40 antibodies. Data were acquired by a FACScan and analyzed by CellQuest Pro software. Histograms represent mean fluorescence intensity. Data shown are representative of one out of five independent experiments.

To determine the effect of M-hMPV protein on APCs, the purified and endotoxin-free prokaryotic recombinant M-hMPV protein was applied into immature moDCs (iDCs). After 24 h of contact with the M-hMPV protein, the phenotype of moDCs was analysed by flow cytometry and by measuring the expression of CD40, CD80, CD83 and CD86 surface molecules. As shown in figure 5, 0.0172 and 0.172 nM of M-hMPV protein were able to induce the up-regulation of CD40, CD80, CD83 and CD86 moDCs cell surface markers in a dose-dependent manner. These results may reflect the capacity of M-hMPV to induce phenotypic maturation of moDCs. The negative controls, such as the HCV-NS3 helicase recombinant protein [24], produced and purified in the same M-hMPV conditions did not change the expression levels of co-stimulatory molecules (data not shown). To further confirm the induction of maturation was by M-hMPV and not through some other mechanism, such as LPS stimulation, moDCs were treated with polymyxin B Sulfate (10 µg/mL) prior treatment with M-hMPV protein and, similarly, moDCs were treated with heat treated M-hMPV protein (100°C for 20 min). Polymyxin B, which prevents LPS activation, did not reduce the M-hMPV protein potential to induce moDCs maturation and an up-regulation of the cell surface markers was still observed. Furthermore, the heat treatment inhibited the ability of M-hMPV to up-regulate the CD40, CD80, CD83 and CD86 cell surface markers and did not induce moDCs maturation (figure 5). M-hMPV treatment did not induce apoptosis of moDCs (Table 1). Taken together, these results suggest that M-hMPV protein can stimulate moDCs into up-regulating their co-stimulatory molecules.

**Table 1:** Percentage of apoptotic M-hMPV-treated moDCs.

	Viable (%)	Apoptotic (%)
iDCs	97	3
LPS 10 ng/mL	82	18
HCV NS3H 0.172 nM	92	8
M-hMPV 0.172 nM	91	9
M-hMPV 0.086 nM	95	5
M-hMPV 0.034 nM	90	10
M-hMPV 0.0172 nM	92	8

After 24 h of stimulation, M-hMPV-treated moDCs were labeled by DiOC6(3) and propidium iodide prior analysis by flow cytometry. Data are representative of one out of two independent experiments.

### M-hMPV induces the production of inflammatory mediators by APCs

**Figure 6:**
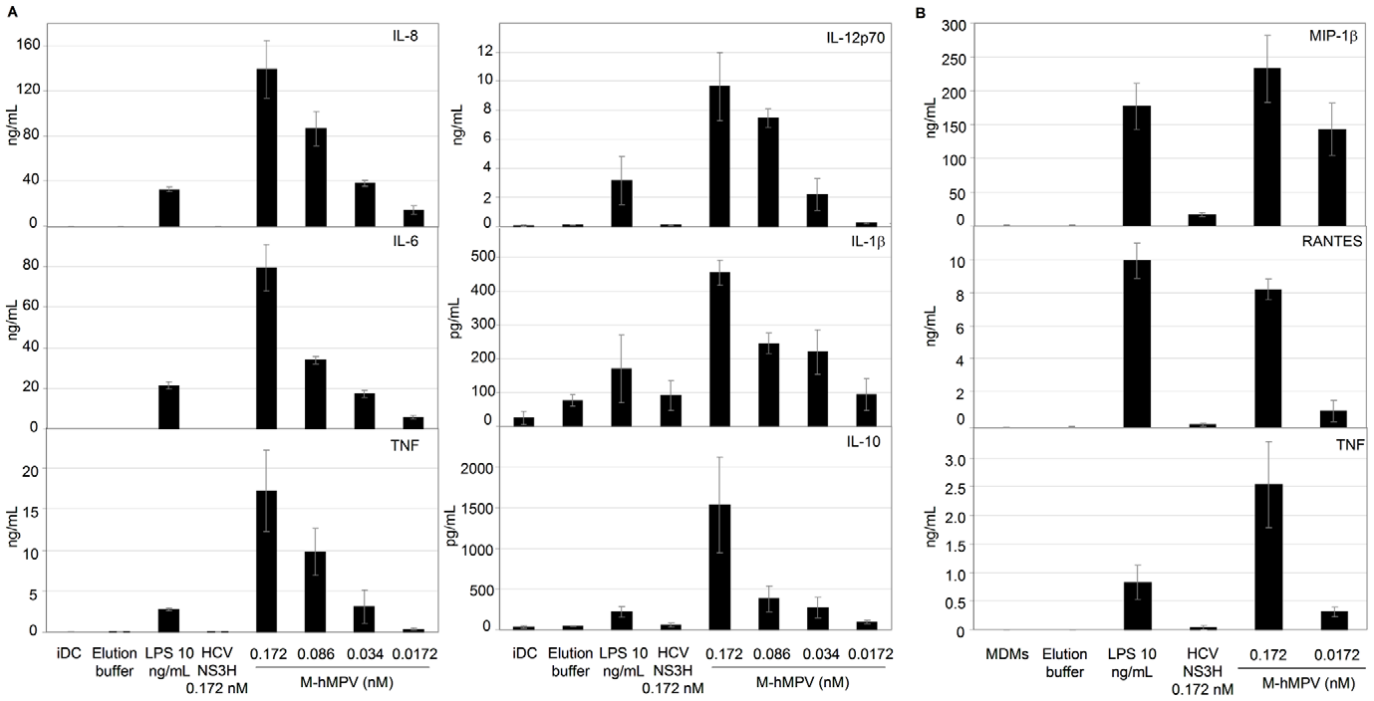
MoDCs and MDMs treated with M-hMPV protein produce high level of cytokines and chemokines respectively. (A) Supernatants of cells treated for 24 h with elution buffer, LPS 10 ng/mL (positive control) and M-hMPV 0.172, 0.086, 0.034, 0.0172 nM were assayed for the level of IL-8, IL-6, TNF, IL-12p70, IL-1β and IL-10 cytokines by CBA. (B) Supernatant of MDMs treated with elution buffer, LPS 10 ng/mL (positive control), M-hMPV 0.172 and 0.0172 nM were assayed for MIP-1β, RANTES and TNF production by CBA. Bar graph represents concentrations values expressed in ng/mL. Data are represented as means of three experiments ± S.D.

In addition to the expression of surface co-stimulatory molecules, the DCs maturation process can lead to the release of soluble mediators, such as cytokines, which affect both humoral and T-cell-mediated immune responses. As shown in figure 6A, M-hMPV induced the production of IL-8, IL-6, TNF, IL-12p70, IL-1β and IL-10 by moDCs in a dose-dependent manner. Furthermore, since chemokines play an important role in the initiation of immune response, we analyzed whether M-hMPV protein impacted on the MDMs chemokine expression profile and on the TNF cytokine. MIP-1β, RANTES and TNF were detected in supernatants of M-hMPV treated-MDMs (figure 6B).

**Figure 7:**
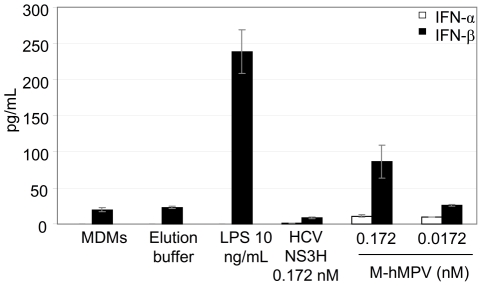
M-hMPV treated-MDMs release low level of IFN-α and IFN-β. MDMs were treated with elution buffer, LPS 10 ng/mL, M-hMPV 0.172 and 0.0172 nM. Supernatants were collected after 24 h and tested for IFN-α and IFN-β production by ELISA. Bar graph represents concentrations values expressed in pg/mL. Data are represented as means of three experiments ± S.D.

M-hMPV induced type I interferon response. The type I interferons (IFNs) play an essential role in the early antiviral cell response. With this in mind, we investigated the potential for M-hMPV to induce these molecules. The secretion of type I IFNs was quantified in MDMs supernatants obtained after 24 h of culture with M-hMPV. M-hMPV treated-MDMs produced only low levels of IFN-β (figure 7), and similarly, little secretion of IFN-α was observed. Furthermore, neither IFN-α, nor IFN-β were detected in the supernatant of M-hMPV-treated moDCs (data not shown).

### M-hMPV induces mature moDCs to activate allogeneic T-cells

**Figure 8:**
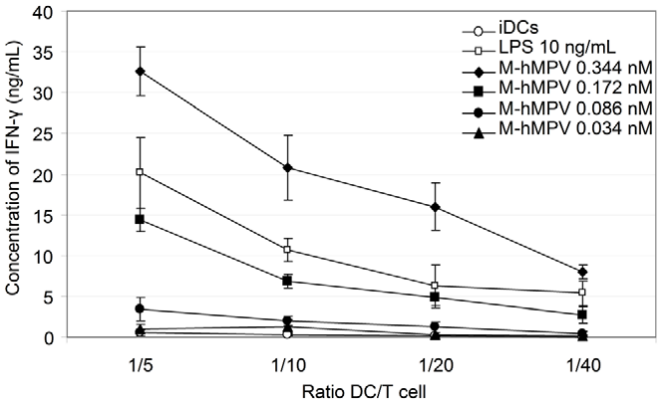
M-hMPV activated-DCs present antigen to T cells which induce the production of IFN-γ. moDCs were treated for 24 h with LPS 10 ng/mL and M-hMPV protein 0.344, 0.172, 0.086, 0.034 nM. Cells were harvested after treatment, washed and cultured for 5 days with allogeneic purified T-cells (2×10^5^/well) at a DC/T ratio ranging between 1∶5 and 1∶40. The amount of IFN-γ in the cell-free supernatants of the co-culture was measured by ELISA. Data are represented as means of three experiments ± S.D.

To further study the maturation level and the functionality of the moDCs, we tested M-hMPV activated DC for their capacity to stimulate the allogeneic T-cells by performing a MLR. The mature moDCs treated with the M-hMPV protein were co-cultured with allogeneic T-cells for either two or five days. Supernatants were recovered and the production of IL-2 (day 2), IFN-γ, IL-5 and IL-13 (day 5) was analyzed. The allogeneic T-cells produced IFN-γ in a ratio- and dose-dependant manner (figure 8). Furthermore, IL-2 was constantly secreted at day 2 and day 5 at about 200 pg/mL (data not shown), but IL-5 and IL-13 were not detected (data not shown).

## Discussion

hMPV is a major cause of respiratory tract illness especially in young children and the need to develop new strategies to deal with such infection remains a priority. The matrix protein is one of the most abundant protein in the virion and its role in the establishment of the immune response is still unknown. The M-hMPV soluble protein was detected in the supernatant of both hMPV infected Vero cells and transiently M-hMPV transfected 293T cells. Because the M-hMPV release has been detected in the supernatant of cells presenting characteristic hMPV cytopathic effects, a hypothesis could be that a process of cell death may occur and allow the release of the matrix protein from infected cells. Further experiments are needed to characterize the release process.

The release from the hMPV infected cells can be the result of a specific mechanism or of cell death. Regarding the transiently M-hMPV transfected 293T cells, most of the protein was released as a soluble form leaving a small proportion of the protein associated with the cell membranes. To our knowledge, this is the first time that a soluble viral matrix protein has been reported. The M-hMPV protein does not have a signal peptide and may not be able to enter the conventional secretion pathway [26]. However, other viral proteins have been described to be secreted without signal peptide, such as HIV tat [27], [28] and HSV VP22 [29]. Moreover, it may undergo secretion through other pathways, such as intracellular vesicular routes (exosomes formation, secretory lysosomes) or other where intermediate intracellular vesicles are not involved (membrane blebbing/shedding, translocation mediated by plasma membrane-resident transporters) [30].

The objective of this study was to determine the M protein effect in the immune response. To further study M-hMPV protein, an endotoxin free M-hMPV recombinant protein was produced in bacteria. In our model, M-hMPV protein demonstrated a dose-dependent binding to moDCs and MDMs along with an efficient and rapid internalization as demonstrated by confocal microscopy. Incubation of moDCs with M-hMPV also led to the up-regulation of co-stimulatory molecules CD40, CD80, CD83 and CD86 indicating that moDCs appeared to be activated and induced to mature. Furthermore, the M-hMPV treatment does not induce apoptosis of the moDCs that could explain the release of cytokines/chemokines. This observed maturation response was unlikely to be caused by LPS contamination as polymyxin B did not affect M-hMPV-induced phenotypic or functional maturation. Heating M-hMPV completely abolished its activation effects whereas LPS is inactivated by heating. Furthermore, M-hMPV protein did not contain detectable nucleic acid contaminants (data not shown) that could activate a Toll-like receptor pathway.

In our *in vitro* model, we observed that moDCs and MDMs treated with M-hMPV were induced to produce cytokines and chemokines associated with their maturation and activation. M-hMPV protein induced a dose-dependent increase of TNF and IL-12p70 production, essential inflammatory cytokines that have been associated with immune responses including Th1-type [31]. The β chemokines RANTES and MIP-1β are implicated in the modulation of different biological process, such as cellular activation, degranulation, and enzyme release [32], [33]. These molecules have also been characterized as the key players in the directional migration of a various number of inflammatory cell types like neutrophils, macrophages, lymphocytes B and T [32], [34], [35]. Thus, cytokines and chemokines produced by activated APCs are indicative of the inflammatory process generated by M-hMPV protein. These data also support the detection of these cytokines by Laham *et al.* [10] in the nasopharyngeal and the tracheal secretion of children infected by hMPV even if the amount of secretion was less important than with RSV. Boivin *et al.* firstly reported an outbreak of hMPV infection in a long-term facility care and presented histopathological findings associated with a fatal pneumonia case in an 89-year-old woman [36]. Immunohistochemical staining of a lung autopsy has been performed by using a monoclonal antibody directed against the hMPV matrix protein and revealed evident localization of the M protein in the cytoplasm of bronchiolar epithelial cells. These observations support that the M-hMPV protein could elicit a persistent innate immune response which may contribute to the pathogenesis of bronchiolitis in infants and young children as well as acute respiratory tract infections in elderly patients infected with hMPV.

The complete maturation of M-hMPV treated-moDCs was demonstrated by their ability to stimulate IFN-γ synthesis by allogeneic T cells indicating that M-hMPV protein is a predominant Th1 promoting innate stimulus. This IFN production is controlled by cytokines secreted by APCs, particularly by IL-12. IFN-γ up-regulates the expression of class I MHC molecules to the cells, which is important for the immune host response as it increases the recognition of pathogens peptides by cytotoxic T cell and induces the cell mediated immunity [37].

The induction of type I IFN molecules is crucial in the establishment of the innate immune response to fight a viral infection [38]. In our experiments, the M-hMPV treated APCs induce a very low quantity of type I IFNs suggesting that the matrix protein did not seem to play a part in the early antiviral immune response against hMPV. Guerrero-Plata *et al.* [39] observed that hMPV infection of moDCs resulted in the release of IFN-α when RSV-infected moDCs failed to produce it. However, both viruses were capable to interfere with IFN-α secretion induced by specific agonists. RSV has been reported as a poor inducer of type I IFN due to the two non-structural proteins NS1 and NS2. Spann *et al.* [40] showed that RSV NS1 and NS2 proteins function independently and co-ordinately to inhibit the IFN production, with NS1 having a greater independent role. hMPV lacks these proteins in its genome but recently, the hMPV glycoprotein G has been identified as a potential inhibitor of the IFN production in airway epithelial cells. Bao *et al.* [41] demonstrated that hMPV is a poor inducer of type I *IFN. The hMPV glycoprotein G has been identified as an important virulence factor that* interacts with and inhibits the activation of RIG-I, an RNA helicase which is involved in the signaling cascade leading to NF-κB, IRF activation and IFN production. The glycoprotein G of hMPV appears to inhibit the production of antiviral molecules like the IFNs. Depending of the cell type, hMPV induces or not a production of type I IFN. It may be of some interest to determine if the M-hMPV protein can act like the glycoprotein G and inhibits the IFNs production by interacting with a sensor of viral infection in DCs.

Tan *et al.* [42] demonstrated that hMPV induces an impaired allo-stimulatory function of dendritic cells, consequently causing a poor production of inflammatory cytokines. In contrast, Guerrero-Plata *et al.* [39] showed that hMPV-infected moDCs did not significantly inhibit T cell proliferation. They also demonstrated that RSV and hMPV differentially activate human DCs. Both viruses were able to mature moDCs characterized by an up-regulation of antigen-presenting and co-stimulatory molecules, however, only RSV-infected moDCs led to impaired T cell activation. This finding has also been observed by de Graaff *et al.* [43] including data demonstrating a failure for T cell activated by RSV-DC to produce IFN-γ. hMPV is a major respiratory pathogen in infants and in the elderly as well as RSV, two genetically distinct members of the *Paramyxoviridae* family that cause indistinguishable clinical symptoms. However, data discussed previously, demonstrate a difference in the immune response. It is interesting that, unlike RSV, hMPV did not stimulate the production of inflammatory cytokines, hMPV-infected DC were able to stimulate T cells [39] but did elicit identical inflammatory symptoms of similar [44] or milder severity [45]. A critical question raised by these studies is the different properties of hMPV isolates used to infect and replicate into APCs. In tissue culture, growth of hMPV is slow and requires the addition of trypsin to the media to efficiently propagate the virus [1], [4]. This difficulty and the adaptation of the virus strain *in vitro* may explain the paradigm of the results obtained by Guerrero-Plata *et al.* and Tan *et al.* in their studies.

The primary defense barriers against pathogens, such as viral particles or proteins of pathogens, are built up by epithelial surfaces in contact with APCs. We hypothesized that hMPV infection in pulmonary epithelial cells may lead to the release of M-hMPV protein stimulating the APCs resident in the respiratory tract. M-hMPV could then selectively and efficiently be processed by APCs. Moreover, M-hMPV induces their maturation, promotes their capacity to stimulate T lymphocytes and finally elicits a predominant Th1 pattern of response.

In conclusion, we demonstrate for the first time that M-hMPV protein can be released in a soluble form and that it can interact with human moDCs and MDMs inducing a response. These M-hMPV/APCs interactions were characterized by (1) the binding and internalization of M-hMPV to human APCs in a dose-dependent manner; (2) the up-regulation of moDCs co-stimulatory molecules CD40, CD80, CD83 and CD86; (3) the secretion of inflammatory mediators IL-8, IL-6, TNF, IL-12p70, IL-1β and IL-10; and (4) the stimulation of T cells by moDCs activated by M-hMPV inducing a predominant Th1 polarization and demonstrating an efficient M-hMPV innate stimulus. These finding suggest that M-hMPV plays a central role in the inflammatory response initiated by the host innate immune system and is highly intertwined in the mechanisms of respiratory pathogenesis.
